# Female cancer survivors: sexual function, psychological distress, and remaining fertility

**DOI:** 10.1007/s10815-024-03051-7

**Published:** 2024-02-21

**Authors:** Elisabeth Reiser, Bettina Böttcher, Charlotte Ossig, Julia Schiller, Susanne Tollinger, Bettina Toth

**Affiliations:** Department of Gynecological Endocrinology and Reproductive Medicine, Medical University of Innsbruck, Anichstrasse 35, 6020 Innsbruck, Austria

**Keywords:** Sexual dysfunction, Depression, Cancer survivor, Fertility preservation, Psychological distress, Menstrual cycle

## Abstract

**Purpose:**

Improved survivorship in cancer patients leads to new challenging issues including potential impairment of quality of life, sexual function, and fertility. The aim of this study was to assess sexual dysfunction (SD) and psychological distress in female cancer survivors who underwent fertility preservation in the past in comparison to reviewed healthy control data from other published studies. Additionally, our focus was on the difference in SD between women with current desire to get pregnant and already completed family planning.

**Methods:**

In this prospective study, 53 female cancer survivors who underwent fertility preservation at time of cancer diagnosis between 2010 and 2020 were invited to a gynecological exam, laboratory assessment, and two questionnaires (Female Sexual Function Index (FSFI) and Hospital anxiety and depression scale (HADS)) in 2022. These scores were compared to results in the literature of healthy controls and depending on anti-Mullerian-hormone (AMH) levels, current desire to have a child, and age.

**Results:**

After a mean follow-up time of 70 ± 50 months, SD was detected in 60.4% (*n* = 32) of the 53 included patients. Normal results regarding HADS-D/anxiety and HADS-D/depression were found in 88.7% and 94.3% of patients, respectively. At time of follow-up, 69.9% (*n* = 40) regained regular menstrual cycles, 52.6% (*n* = 20) < 40 years showed a diminished ovarian reserve with AMH levels < 1.1 ng/ml and 28.3% (*n* = 15) suffered from infertility.

**Conclusion:**

Female cancer survivors may be at risk for SD. Cancer patients should be informed about possible sexual dysfunction already at the start of cancer treatment and during follow-up. In addition, contraception needs to be addressed if regular cycles occur as more than two-thirds of the women regained regular menstrual cycles.

**Supplementary Information:**

The online version contains supplementary material available at 10.1007/s10815-024-03051-7.

## Introduction

With earlier diagnosis and improved cancer treatment, the group of cancer survivors is constantly rising: while the 5-year survival rate for all cancers combined was 39% in the 1960s, it has increased substantially to 68% in 2021 [1]. With regard to breast cancer, the most common cancer type in women, the 5- and 10-year relative survival rates are as high as 90% and 84%, respectively [1].

Chemotherapy, radiation therapy, hormonal interventions, and surgical procedures may disrupt the delicate balance of reproductive organs, hormonal systems, and neural pathways, leading to a range of challenges in sexual health and fertility. Patients are not only facing short-term consequences but also challenging issues such as comorbidities resulting from the cancer treatment itself, secondary malignancy, and potential impairment of quality of life, sexual function, and fertility. Sexual dysfunction (SD) is a common disease in the general population with a prevalence of 40% in the USA [2]. In female childhood cancer survivors, SD ranges from 20 to 52% and includes problems such as low interest, vaginal dryness, vaginal pain/ discomfort, and difficulties enjoying sex [3]. The prevalence of SD in adult cancer patients seems to be even higher [4, 5]. To date, the Female Sexual Function Index (FSFI) seems to be the only instrument validated both in the general population and in cancer survivors [6, 7].

The life-threatening diagnosis of cancer afflicts the patients’ psychological constitution not only during the acute phase of diagnosis and treatment, but also during and after recovery [8]. Psychological distress affects up to 55% of gynecological cancer patients and seems to have a negative effect on quality of life, cancer recovery, a higher risk of suicide feelings, and long-term survival [9, 10]. Psychological distress such as anxiety and depression are commonly measured by the Hospital Anxiety and Depression Scale (HADS), and its accuracy has been proven in cancer patients [11].

Contrary to investigating only possible negative factors on cancer survivors, it was revealed that positive affect is associated with longer survival [12]. The aim of this study was to assess SD and psychological distress in female cancer survivors who underwent fertility preservation. We were interested in identifying potential risk factors associated with SD, in general and especially in comparing patients with completed family planning with patients with a desire to have children. The desire to get pregnant and its potential risk of sterility may influence sexual function. Impaired sexual functioning in patients suffering from sterility independent of former cancer disease has been shown in the literature [13]. Ovarian reserve is a limiting factor in family planning, especially in cancer survivors. As anti-Müllerian hormone (AMH) is the most established marker for ovarian reserve [14], we chose AMH as a further potential risk factor for SD/depression/anxiety. Existing literature focuses on differences in sexual function/depression/anxiety depending on cancer treatment, type of cancer, time since diagnosis.

## Materials and methods

### Patients

Fertility preservation (FP) has been performed at the Department of Gynecological Endocrinology and Reproductive Medicine, Medical University of Innsbruck, Austria, for over 20 years. All currently available and recommended treatment options are offered. Between 2010 and 2020, 285 female patients were counseled and 255 underwent FP. In order to conduct the present study, all patients were informed by letter, e-Mail, or phone call about the study and invited to participate in a follow-up visit between June and November 2021. Informed consent was obtained at the follow-up visit. Ethical approval was granted by the institutional review board (approval number 1471/2020).

### Measures

Data on demographics, type of cancer, cancer treatment, and FP options were obtained. FP methods included cryopreservation of ovarian tissue, ovarian stimulation with cryopreservation of fertilized and non-fertilized oocytes, transposition of the ovaries, and GnRH analogues. Hormone analysis included anti-Müllerian hormone (AMH, Elecsys® Roche), follicle-stimulating hormone (FSH, ECLIA® Roche), and estradiol (ECLIA® Roche) concentrations at time of FP and follow-up (mean time of follow-up: 70 ± 50 months). Patients underwent transvaginal ultrasound at time of FP and follow-up to estimate antral follicle count (AFC). Patients were asked about their menstrual cycle. A regular menstrual cycle was defined as a menstrual cycle length between 24 and 35 days.

### Questionnaires

To assess SD, the Female Sexual Function Index (FSFI) was applied (Supplemental S1). The questionnaire comprises six domains: desire (two items), arousal (four items), lubrication (four items), orgasm, satisfaction, and pain (three items each). Each individual domain score needs to be multiplied by the domain factor. The FSFI was originally developed and validated in healthy women [15]. Baser et al. evaluated the psychometric validity of the FSFI in cancer survivors and demonstrated that the FSFI was also suitable for monitoring sexual function and dysfunction in sexually active women who underwent cancer treatment with(out) chemo- and/or radiotherapy [16]. Therefore, the FSFI was chosen for this study population. Communal et al. graduated the total score into three grades: normal sexual function (30–36 points), moderate dysfunction (23–29 points), and severe dysfunction (2–22 points) on the basis of results of Rosen et al., with an average full FSFI score of 30 [15, 17]. Data of the general population were available also from the study by Rosen et al. and Lammerink et al. [15, 17]. Our results were compared to the median values of the FSFI of the 119 patients between 30 and 40 years to rule out possible bias of age difference. Wiegel et al. set a cut-off value of 26.55 to distinguish between women with and without SD [18]. Therefore, the cut-off of 26.55 was applied to our study population to identify those with SD.

The Hospital Anxiety and Depression Scale (HADS) was originally designed to screen for anxiety and depression in non-psychiatric hospital clinics [19]. It is the most frequently applied psychometric instrument for psychological distress screening [20] and also scored highest compared to other instruments in the evaluation by Luckett et al.[21]. It is a 14-item questionnaire with cutoff points indicating “cases” helping clinicians to decide whether or not a mental health care professional should be integrated in the treatment of a patient. Each item of the HADS has a Likert response scale. Scores are constructed by summation, whereby increasing scores indicate increasing burden. There are two subscales: depression (HADS-D) and anxiety (HADS-A). The anxiety scale records symptoms such as general worry or nervousness in addition to a generalized anxiety disorder. The depression scale includes various symptoms of a depressive episode, including a reduction in drive and a loss of interest for life, and motivation. Each subscale consists of seven questions with four answer options, allowing a score between 0 and 3. The higher the score, the more pronounced the symptoms. Accordingly, a point value of 0–21 results for each scale, giving a maximum achievable total score of 42 points. The cut-off values defined by the authors who developed the questionnaire allow patients to be assigned to the groups “non-cases” (0–7 points), “suspicious cases” (8–10 points), and “conspicuous cases” (≥ 11 points) (Supplemental S2). Results were compared to a female norm sample by Hinz and Brähler et al. [22] and to the accuracy analysis of Annunziata et al. where another optimal cut-off was described: > 9 units for the HADS-A and > 7 units for the HADS-D [11].

### Statistics

The statistical analysis was conducted using IBM SPSS Statistics for Windows, version 26.0 (IBM Corp., Armonk, NY, USA). Analysis of variance (ANOVA) was performed for normally distributed raw data, which was presented as mean ± standard deviation (s.d.). For non-normal data distribution, the differences between the individual parameters of the groups were analyzed using the Kruskal–Wallis or Mann–Whitney *U* test and presented as median (interquartile range [IQR]). To prevent alpha-error accumulation, Bonferroni correction was applied for multiple comparisons. The Spearman’s rank correlation analysis was used to identify correlations between different parameters. After analyzing the study population with regard to the desire to have children, patients were divided into two groups (current desire vs no desire to have children) in order to further analyze the data of the subgroup “current desire to have children” in particular. Furthermore, different age groups at time of follow-up (< 40 years vs. ≥ 40 years) were analyzed. Association between dichotomous variables was analyzed with the chi square test. A significance level of *α* = 0.05 was selected for all statistical evaluations.

## Results

**Fig. 1:**
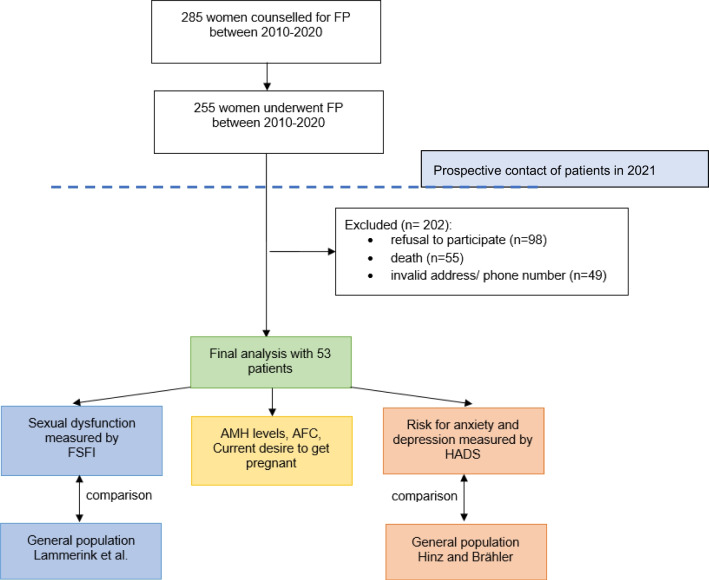
Flow chart of the study design and participants

The majority of patients (*n* = 202) did not participate due to refusal (*n* = 98), death (*n* = 55), or invalid address/phone number (*n* = 49). However, 53 patients were included in the study between June and November 2021. A flow chart of the study design is shown in Fig. 1. Demographic data are displayed in Table 1. Mean age at diagnosis and at time of follow-up was 28.3 ± 6.9 and 33.4 ± 7.1 years, respectively, with a mean follow-up time of 70 ± 50 months.

**Table 1:** Patients’ characteristics

Parameter	*n* = 53 (%)
Type of cancer	
Breast cancer	25 (47.2)
Hematological Malignancy	23 (43.4)
Sarcoma	5 (9.4)
Age at diagnosis (years) (mean, SD)	28.3 ± 6.9
Age at follow-up (years) (mean, SD)	33.4 ± 7.1
Follow-up time in months (mean, SD)	70 ± 50
Gonadotoxic therapy	
Chemotherapy	53 (100)
Combined treatment (+ Radiotherapy)	29 (54.7)
Fertility preservation method	
Cryopreservation of oocytes	26 (49.1)
Cryopreservation of embryos	15 (28.3)
Ovarian tissue banking	36 (62.3)
GnRH-analogues	50 (94.3)
AMH at time of diagnosis in ng/ml (median, IQR)	2.07 (1.07–4.69)
AMH at follow-up in ng/ml (median, IQR)	0.64 (0.08–1.50)
AFC at follow-up (mean, SD)	6.4 $\pm$ 4.1

*AMH* anti-Mullerian-hormone, *AFC* antral follicle count, *SD* standard deviation, *IQR* interquartile range

With regard to cancer treatment, 2 (3.8%), 37 (69.8%), and 14 (26.4%) patients received chemotherapy of low, intermediate, and high risk, respectively. Median AMH levels at time of diagnosis and at time of follow-up were 2.07 ng/ml (1.07–4.69) and 0.64 ng/ml (0.08–1.50), respectively (*p* < 0.001). AMH levels were < 1.1 ng/ml in *n* = 20 (52.6%) patients < 40 years and in 15 (46.7%) patients with current desire to become pregnant. Most of the patients (*n* = 40 (69.9%)) returned to a regular menstrual cycle after the end of gonadotoxic treatment. Mean AFC was 6.4 ± 4.1 at time of follow-up.

### Fertility

The main goal of fertility preservation is to give cancer patients the chance to have children after overcoming their disease. Therefore, we compared patients with desire to get pregnant to patients with completed family planning.

At time of follow-up, 13 (24.5%) patients had their family planning completed, 25 (47.2%) did not have a current desire to have a child, and 15 (28.3%) suffered from infertility. In total, 12 patients (*n* = 1 woman with 3 and *n* = 2 women with two pregnancies) achieved a pregnancy after gonadotoxic treatment with a live birth rate of 62.5%. Out of the *n* = 16 observed pregnancies, 11 were naturally conceived. The other five pregnancies were achieved by embryo transfer with a cryopreserved embryo before gonadotoxic treatment and resulted in three live births. Mean age of patients with infertility at time of diagnosis was 34 ± 5.8 years, and median AMH level was 1.08 (0.35–1.69) ng/ml.

### Sexual dysfunction

**Fig. 2:**
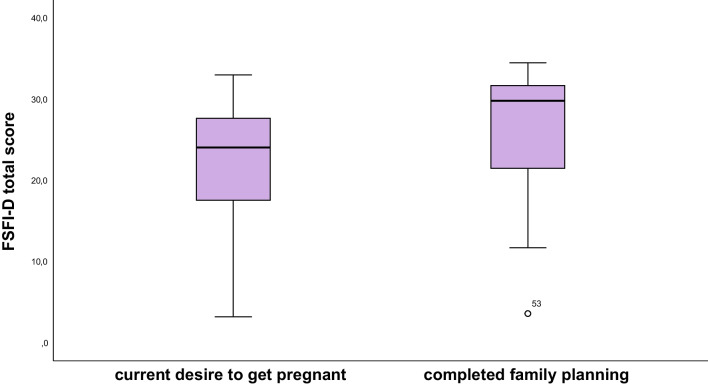
Boxplot of FSFI-D score in women with current desire to get pregnant compared to women with completed family planning

All patients were sexually active. Results of the single domains and the comparison with the general population of Lamnerink et al. are shown in Table 2. In all domains tested, including desire, arousal, lubrification, orgasm, satisfaction, and pain, study patients, both women with current desire to get pregnant and already completed family planning, reached significantly lower scores compared to the general population of Lamnerink et al. as illustrated in Table 2 [23]. Mean and median FSFI-D overall score was 22.9 (± 8.6) and 24.4 (17.9–29.9), respectively. In all domains, study patients reached significantly lower scores compared to the general population [23]. When applying the cut-off value by Wiegel et al., 32 (60.4%) patients suffered from SD. With regard to the differentiation by Communal et al., severe, moderate, and no SD was present in 20 (37.7%), 17 (32.1%), and 16 (30.2%) patients, respectively. No significant association was found between SD and AMH levels, type of cancer (hematological vs. breast cancer), or age (*p* = 0.49, *p* = 0.56, and *p* = 0.36). Median FSFI-D score of women with current desire to get pregnant and already completed family planning was 21.1 (7.2–27.5) and 25.4 (20.3–21.4), respectively (*p* = 0.065) (Fig. 2).

**Table 2:** FSFI domains compared to the general population

Domain	*n*	Mean (SD)	Median	*p* value
Desire				
Follow-up	53	3.0 (1.2)	3.0 (2.4–3.6)	0.010*
General population^a^	119	3.5 (1.0)	3.6 (1.8–5.4)	
Arousal				
Follow-up	53	3.9 (1.7)	3.9 (3.0–5.4)	< 0.001*
General population^a^	119	5.2 (0.8)	5.4 (3.3–6.0)	
Lubrication				
Follow-up	53	4.2 (2.0)	4.8 (3.3–6.0)	< 0.001*
General population^a^	119	5.6 (0.7)	6.0 (3.9–6.0)	
Orgasm				
Follow-up	53	3.9 (1.9)	4.0 (2.4–6.0)	< 0.001*
General population^a^	119	5.0 (1.3)	5.6 (2.0–6.0)	
Satisfaction				
Follow-up	53	4.0 (1.7)	4.0 (2.6–5.6)	< 0.001*
General population^a^	119	5.1 (1.0)	5.2 (2.8–6.0)	
Pain				
Follow-up	53	3.8 (2.3)	4.8 (2.8–5.6)	< 0.001*
General population^a^	119	5.6 (0.9)	6.0 (3.2–6.0)	

^a^Data available from Lammerink et al. [23]

There was no significant difference in SD between these two groups (*p* = 0.69).

### Psychological distress

**Fig. 3:**
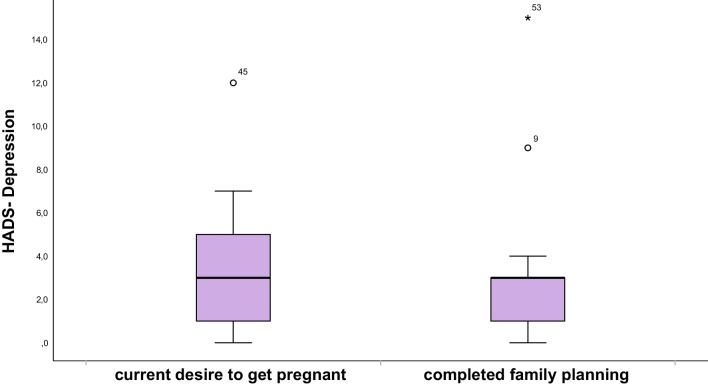
Boxplot of HADS-D/Depression score in women with current desire to get pregnant compared to women with completed family planning

**Fig. 4:**
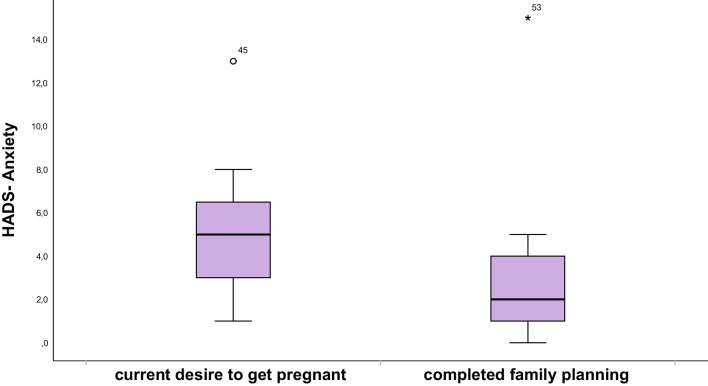
Boxplot of HADS-D/Anxiety score in women with current desire to get pregnant compared to women with completed family planning

Next, psychological distress was evaluated and mean total score, anxiety, and depression sub scores were 7.7 (± 5.5), 4.4 (± 2.9), and 3.3 (± 2.9), respectively. Normal HADS-D/A and HADS-D/D results were seen in 47 (88.7%) and 50 (94.3%) of patients, respectively (Table 3). Thus, the scores of patients after gonadotoxic therapy are slightly lower than those of the general population (comparison to the female norm sample by Hinz and Brähler et al. [22]) and do not provide evidence of increased rates of anxiety or depression. Same results were achieved when applying the cut off by Annunziata et al. [11]. Compared to patients with completed family planning, women with current desire to get pregnant showed higher risk for both depression and anxiety (Figs. 3 and 4), although not reaching statistical significance (0.75 and 0.29).

**Table 3:** Application of different cut off values on HADS-D/Anxiety and HADS-D/Depression results

HADS-D-Groups	*n* (%)	Mean (SD)	Median
HADS-D/A	53	4.42 (2.9)	4.0 (2.0–6.0)
Normal (0–7 points)^a^	47 (88.7)		
Suspicious (8–10 points)^a^	4 (7.5)		
Conspicuous (≥ 11 points)^a^	2 (3.8)		
Normal (< 9 points)^b^	49 (92.5)		
Suspicious (> 9 points)^b^	4 (7.5)		
HADS-D/D	53	3.34 (2.9)	3.0 (1.0–5.0)
Normal (0–7 points)^a^	50 (94.3)		
Suspicious (8–10 points)^a^	1 (1.9)		
Conspicuous (≥ 11 points)^a^	2 (3.8)		
Normal (< 7 points)^b^	50 (94.3)		
Suspicious (> 7 points)^b^	3 (5.7)		

^a^Cut off in accordance to [22]

^b^Cut off in accordance to [11]

## Discussion

In the present study, we found a high prevalence of SD in female cancer survivors who underwent fertility preservation in the past, especially general SD (60.4%). This percentage is nearly twice as high as in a healthy population: 37.7% suffer from severe and 32.1% from moderate SD. However, the risk for psychological distress (anxiety and depression) was not significantly increased in cancer survivors. Most patients have not completed their family planning at time of follow-up and showed signs of a diminished ovarian reserve with low AMH levels and reduced AFC.

Our findings with regard to SD are in line with other studies. In a review including 20 different cancer types, mean FSFI value ranged from 16.25 to 19.58 in middle-aged patients with colorectal, gynecological, and breast cancer, respectively. SD prevalence was higher than 60% in all cancer types, with the highest value for gynecological cancer (78.44%, 95% CI 68.36–88.52%) [24]. When aged-matched cancer survivors were analyzed (mean age at follow-up, 29 years) with a longer follow-up period (mean 20.9 (± 7.8 years), SD was present in 57% [3]. Dysfunction was most common for sexual interest (36%), orgasm-ability (32%), and vulvar discomfort (19%). In our study, dysfunction was most common in orgasm, arousal, desire, and satisfaction. Of note, SD was assessed by another tool—the PROMIS Sexual Function and Satisfaction—but with similar domains.

Studies focusing on breast cancer patients found a prevalence of SD of 75% in 64 patients assessed by the FSFI and applying the cut-off value of 26.55 [20]. In addition, an association between sexual function and age, hormone therapy, and women’s mental health was detected. Hormone therapies (e.g., tamoxifen and aromatase inhibitors) can lead to vaginal dryness and thus changes in neurobiological function such as desire or arousal affecting also orgasm. In our study, 30% of women were under ongoing hormone therapy at the time of follow-up. Although age did not seem to influence the prevalence of SD in our study, data from previous studies imply that sexual satisfaction, lubrication, sexual arousal, and frequency of sexual intercourse decrease physiologically with increasing age [25, 26]. Moreover, in women with breast cancer, sensation in the affected breast may be altered, possible painful scars, and a constant reminder of the disease triggered by touching the breast could affect sexuality. After breast surgery, self-esteem might be lower, and feelings of shame or not feeling as attractive as before may occur and have an impact on sexuality. Additionally, although more than half (69%) of our patients had a regular menstrual cycle, 26% reported amenorrhea, which has a significant impact on sexual function by causing vaginal dryness [25]. Possible influencing factors include duration and type of gonadotoxic treatment as well as age at treatment and age at time of follow-up. In breast cancer patients, chemo- and radiotherapy duration is usually 6–8 months, not including adjuvant therapies such as tamoxifen and aromatase inhibitors. In hematological cancer patients, treatment duration lasts between 8 and 10 months. Patients were at a young mean age at time of treatment (28.3 years) and time of follow-up (33.4 years) with an adequate follow-up time in which return of menstrual cycle is possible. In this young cohort, a prevalence of amenorrhea of 26% is high as side effects/consequences of an estrogen deficiency including risk of osteopenia, osteoporosis, cognitive impairment, and coronary artery disease need to be prevented.

The prevalence of anxiety and depression in cancer patients in the literature ranges from 7 to 88% and from 3 to 65.5%, respectively [20]. In our study, risk for anxiety and depression was 11.3% and 5.7%, respectively. Possible risk factors as detected in the review by Ostovar et al. include cancer type (breast and lung cancer), cancer stage (advanced), gender (female), age (older-aged), and type of therapy (chemotherapy). Of note, 16 different screening tools were used in the included studies [20]. Especially patients with advanced breast cancer seem to suffer from anxiety and depression [27]. Focusing on the cohort of female cancer survivors with metastatic breast cancer < 40 years, Park et al. found mean scores of 4.4 ± 3.7 and 7.9 ± 5.0 in the HADS depression and HADS anxiety scale, respectively [28]. In both subscales, especially in the anxiety subscale, these results are higher than in our cohort which might be linked to the metastatic situation. In patients with hematologic cancer, the other major part of our cohort, anxiety, and depression is less frequently investigated in long-term survivors. In early survivorship, anxiety seems not to be influenced by unmet psychological needs during chemotherapy [29]. In a Swedish study, 20 out of 54 participants (37%) showed a higher score in the anxiety subscale than the control group (HADS questionnaire) [30]. On the other hand, the depression scale revealed only 8% participants with depressive symptoms. Interestingly, since anxiety and depression are not increased in our cohort and all patients are sexually active, the likelihood of a sole psychological explanation is limited and might be explained by increased resilience after the survival of cancer and the lack of disease recurrence.

According to the literature, AMH might reflect remaining ovarian reserve after cancer treatment [31, 32]. Women with low AMH levels (< 0.7 ng/ml) and thereafter diminished ovarian reserve seem to have a lower chance to achieve a pregnancy [33]. In our study, median AMH levels were 1.07 ng/ml, and 53% of the participants younger than 40 years showed values below 1.0 ng/ml at time of follow-up. However, no association between lower AMH values and increased risk for anxiety, depression, or SD was found.

When focusing on infertile women, Tanha et al. found impaired sexual function in infertile women (25.7 ± 4.6 years) compared to fertile women (32 ± 1.1 years) by applying the FSFI [34]. Also, in our study, 11/15 patients with current desire to get pregnant suffer from SD, compared to only 5/13 who already completed their family planning.

### Study limitations

Although our results reflect recently published date, there are some limitations. First, the low participation rate may have led to a selection bias. Patients who are willing to participate in a study might be in better psychological and sexual functioning state with less prevalence for depression and anxiety. Due to the small sample size, we were not able to control for confounding factors, such as medication or ongoing psychotherapy. Second, the relatively long study interval ended up in the loss of more than 50 patients. Third is the heterogeneity of our cohort with regard to cancer type, chemotherapy regime, and chosen fertility preservation method. Fourth is the single time point of the questionnaire. In addition, other questionnaires, such as the Short-Form-36 (SF-36) or the Sense of Coherence-13 (SOC-13) might provide a more comprehensive picture of patients’ mental health [30].

### Clinical implications

As SD is present in young female cancer survivors, sexual function needs to be addressed at the time of fertility preservation counseling and during follow-up visits. Possible approaches especially in adolescent and young cancer survivors are displayed in the review by Peleg Nesher et al. [35]: after evaluation of SD in four steps as provided by Bartlik et al. [36], patients can be offered couple/sex therapy, sexual medicine or further referral to a specialist (gynecologist/endocrinologist/urologist). Depending on current family planning, both contraception and hormone therapy as well as non-hormonal treatment options can be offered to improve vaginal dryness and other treatment-related discomfort.

## Conclusions

In conclusion, female cancer survivors may be at increased risk for SD. Thereafter, sexual function should be discussed with both: the patient and the partner. As two-thirds of our patients regain a regular menstrual cycle, contraception also needs to be addressed. Patients with persistent amenorrhea should also be counseled concerning the option of hormone therapy. The fact that risk for depression and anxiety is not increased in our cohort as a possible sign for the achievement to find back to normal life needs to be interpreted with caution (as do all of our study findings) due to the small sample size. Follow-up of cancer patients is of utmost importance to address these concerns.

### Supplementary Information

Below is the link to the electronic supplementary material.

Supplementary file1 (DOCX 14 KB)

## Data Availability

Data is unavailable due to privacy.
