# Supplementary material for: Female cancer survivors: sexual function, psychological distress, and remaining fertility
Source: J Assist Reprod Genet. 2024 Feb 21;41(4):1057–65. doi: 10.1007/s10815-024-03051-7 (PMC11052740; doi:10.1007/s10815-024-03051-7)
Supplement: Supplementary file 1 — Supplementary file1 (DOCX 14 KB) [file 10815_2024_3051_MOESM1_ESM.docx]

Supplemental Material.

**Table S1. Female Sexual Function Index (FSFI): Domain and Scoring**

| **Domain** | **Questions** | **Score Range** | **Factor** | **Minimum Score** | **Maximum Score** |
| --- | --- | --- | --- | --- | --- |
|  |  |  |  |  |  |
| Desire | 1 - 2 | 0 -5 | 0.6 | 1,2 | 6 |
| Arrousal | 3 -6 | 0 -5 | 0.3 | 0 | 6 |
| Lubrication | 7 -10 | 0 -5 | 0.3 | 0 | 6 |
| Orgasm | 11 -13 | 0 -5 | 0.4 | 0 | 6 |
| Satisfication | 14 – 16 | 0 -5 | 0.4 | 0.8 | 6 |
| Pain | 17-19 | 0 – 5 | 0.4 | 0 | 6 |
|  |  |  |  |  |  |
| Full Scale Score Range |  |  |  | 2 | 36 |

**Table S2. Classification of Hospital anxiety and depression scale (HADS) by Hinz and Baehler (17)**

| **HADS-D-Depression scale** |  |
| --- | --- |
|  |  |
| 0 – 7 points | Non-cases: high likelihood of no present depressive disorder |
| 8 – 10 points | Suspicious cases: possible presence of a depressive disorder |
| ≥ 11 points | Conspicuous Cases: severe depressive symptoms, high likelihood of a present depressive disorder |
| **HADS-D-anxiety scale** |  |
| 0 – 7 points | Non-cases: high likelihood of no present anxiety disorder |
| 8 – 10 points | Suspicious cases: possible presence of a anxiety disorder |
| ≥ 11 points | Conspicuous: severe symptoms of anxiety, high likelihood of a present anxiety disorder |
| **HADS-D total score** |  |
| ≥ 13 points | doubtful: possible presence of a depressive and/or anxiety disorder |
| ≥ 15 points | cases: high likelihood of a present depressive and/or anxiety disorder |
